# Dental Intervention on the Quality of Life of Metabolic Syndrome Patients: A Randomized Controlled Trial

**DOI:** 10.3390/jcm15072788

**Published:** 2026-04-07

**Authors:** Sahaprom Namano, Yuriko Komagamine, Bui Ngoc Huyen Trang, Maiko Iwaki, Kaho Hoteiya, Terumi Sakaguchi, Shunsuke Minakuchi, Manabu Kanazawa

**Affiliations:** 1 Department of Gerodontology and Oral Rehabilitation, Graduate School of Medical and Dental Sciences, Institute of Science Tokyo, Tokyo 113-8510, Japan; sahaprom.n@chula.ac.th (S.N.); bnhtrang@ntt.edu.vn (B.N.H.T.); hoteiya.kaho@tmd.ac.jp (K.H.); s.minakuchi.gerd@tmd.ac.jp (S.M.);; 2 Division of Academic Affairs, Faculty of Dentistry, Chulalongkorn University, Bangkok 10330, Thailand; 3 Prosthodontics Division, Department of Restorative Sciences & Biomaterials, Boston University Henry M. Goldman School of Dental Medicine, Boston, MA 02118, USA; 4 Department of Prosthodontics and Maxillofacial Prosthetics, Faculty of Dentistry, Nguyen Tat Thanh University, Ho Chi Minh City 700000, Vietnam; 5 Digital Dentistry, Graduate School of Medical and Dental Sciences, Institute of Science Tokyo, Tokyo 152-8550, Japan; m.iwaki.gerd@tmd.ac.jp

**Keywords:** dental intervention, metabolic syndrome, obesity, oral health-related quality of life, periodontal treatment, prosthodontic treatment

## Abstract

**Background/Objectives**: Metabolic syndrome (MetS) causes significant oral manifestations that negatively impact oral health-related quality of life (OHRQoL). This randomized controlled trial evaluated the effects of combined dental interventions and lifestyle guidance on OHRQoL in patients with MetS. **Methods**: In total, 82 participants with MetS were randomized into an intervention group (IG; *n* = 39), receiving dental treatment plus lifestyle guidance, or a control group (CG; *n* = 43), receiving lifestyle guidance only. OHRQoL was assessed using GOHAI and OHIP-14 at baseline, 1 month, and 3 months. Data were analyzed using repeated-measures ANOVA and multivariable ANCOVA, adjusting for age, sex, baseline OHRQoL, and waist circumference. Pearson correlations examined the relationship between metabolic changes (Δ) and OHRQoL. **Results**: At 3 months, the IG demonstrated significantly superior OHIP-14 scores (*p* = 0.020) and a large effect size in social disability ($\eta_{p}^{2}$ = 0.148, *p* < 0.001) compared to the CG. Within-group analysis showed the IG achieved highly significant longitudinal improvements in pain and psychological discomfort (all *p* < 0.001). Subgroup analysis confirmed these gains were primarily driven by participants with missing teeth ($\eta_{p}^{2}$ = 0.447, *p* < 0.001), whereas the periodontitis-only subgroup showed non-significant shifts. Multivariable analysis identified age and baseline scores as primary predictors. Notably, OHRQoL improvements significantly correlated with reductions in body weight (r = 0.355, *p* = 0.001) and waist circumference (r = 0.238, *p* = 0.031). **Conclusions**: Integrated dental and lifestyle interventions significantly improved OHRQoL in MetS patients by enhancing psychosocial well-being and social reintegration. Gains were functionally driven by systemic metabolic success. Addressing “nutritional barriers” through dental rehabilitation, while targeting weight loss goals, was essential for holistic MetS management.

## 1. Introduction

Metabolic syndrome (MetS) [1], a global health crisis, has increased the risk of type 2 diabetes and cardiovascular disease, while increasing healthcare costs by 20% [2]. MetS affects roughly 25% of the global population; however, prevalence rates fluctuate depending on the diagnostic criteria applied and demographic factors such as age and ethnicity [3,4]. In Japan, an estimated 19.4 million (15.3%) nationals aged 40–74 years are affected [5]. The current Japanese MetS criteria [6] are based on the third revision of the Adult Treatment Panel of the National Cholesterol Education Program (NCEP-ATP III) [7], which defines MetS as abdominal obesity accompanied by diabetes, hypertension, or dyslipidemia.

MetS negatively impacts health-related quality of life (QoL; HRQoL) [8,9]. Dental problems can also affect physical function, social relationships, and oral health-related QoL (OHRQoL) [10,11]. De Santoso et al. discovered a substantial association between MetS and poor OHRQoL [12]: MetS-induced inflammatory and oxidative stress might have impacted oral health, causing poor OHRQoL. Previous studies examined the association between MetS and oral problems, where the majority showed that periodontal disease-induced inflammation may have contributed to MetS, causing insulin resistance and dyslipidemia [12,13,14]. Tooth loss can affect eating choices and nutritional intake, causing metabolic abnormalities [15,16,17]; however, while the link between MetS and oral problems is well-established, the impact of MetS on OHRQoL remains poorly understood.

Because metabolic syndrome is associated with chronic systemic inflammation, it may also influence oral conditions such as periodontal disease, which can negatively affect oral health-related quality of life [4]. Intervention studies on periodontal therapy impacting glycemic control in individuals with diabetes and periodontal disease have typically revealed a favorable effect, with lower glycated hemoglobin levels [18,19,20]. Periodontal and prosthodontic treatment can successfully regulate glycemia in diabetic individuals [21]. Saengtipbovorn and Taneepanichskul performed a clinical experiment in which individuals with diabetes received both lifestyle counseling and dental treatment [22]. While prior publications have primarily focused on dental treatment as a standalone intervention, this study uniquely combined dental care with lifestyle therapy. However, to date, no studies (or only an extremely limited number of studies) have investigated the effect of an intervention combining dental treatment and lifestyle guidance on OHRQoL among patients with MetS. Therefore, the aim of this randomized controlled trial was to evaluate whether dental treatment combined with lifestyle guidance could improve oral health-related quality of life among patients with metabolic syndrome [23]. The null hypothesis is that dental treatments in MetS patients would negatively affect self-reported OHRQoL.

## 2. Patients and Methods

### 2.1. Patients

The trial protocol was approved by the Faculty of Dentistry’s Ethics Committee at the Tokyo Medical and Dental University (TMDU) (Registration No. D2016-028, approved on 6 December 2016, final registration date 25 January 2019). The University Hospital Medical Information Network Centre (UMIN-CTR Clinical Trial, UMIN000022753) included the trial registration.

Informed consent was obtained from all individual participants included in the study. The included participants were TMDU workers and patients aged at least 40 years who met the MetS waist circumference criteria, had missing teeth, or had light-to-moderate periodontitis (4 mm ≤ pocket depth < 6 mm) [24]. To diagnose missing teeth, Eichner’s criteria were used: A2, A3, B1, B2, B3, B4, C1, C2, and C3, with “A” indicating full support; “B,” partial support; and “C,” no opposition [2]. The exclusion criteria included external lifestyle guidance, compliance barriers, medication changes within 3 months, pregnancy, or severe systemic diseases (cardiovascular, thyroid, hepatic, renal, or malignancy). Doke et al. provided a sample size estimation of 50 participants in each group and a flowchart of the baseline characteristics of the included and excluded patients [23].

A single institution (TMDU Dental Hospital) conducted this double-blind, parallel-group randomized controlled trial (RCT), employing sealed opaque silver-paper-lined envelopes to allocate the participants into two groups: intervention (IG) and control (CG). In this double-blind RCT, participants and operators were blinded to these groups, while the coordinator remained unblinded to perform allocation and assessment. Due to the nature of the dental interventions, it was not possible to blind the clinical staff or the participants to the group allocation. The study coordinator, who managed the assessments, was also unblinded. However, to reduce the risk of detection bias, OHRQoL data were collected via self-reported, standardized questionnaires (GOHAI and OHIP-14), which participants completed independently to minimize external influence. Waist circumference differences > 3.0 cm (standard deviation: 5.0 cm) within both groups were regarded as statistically significant. Because no prior clinical significance threshold for OHRQoL existed, significance was determined through statistical analysis.

Participants who underwent baseline examinations received dietary and exercise guidance, or so-called lifestyle guidance. Lifestyle guidance was provided using the Total Fitness Analysis System (TFAS), which evaluated lifestyle behaviors and provided individualized health recommendations [25,26,27,28]. After inputting their recent dietary and exercise records, the participants received personalized results and feedback through the TFAS and were instructed to attend a 60 min video lecture conducted by a health science specialist that aimed to boost motivation for health improvement without enforcing specific diets or exercises. The initial Specific Health Guidance [29] session lasted a maximum of 80 min, ensuring a comparable trial intensity.

Regarding dental treatments, all participants required periodontal treatment at baseline, and some required dental prostheses. The IG received lifestyle guidance plus nonsurgical periodontal treatment (oral hygiene instruction, supragingival scrubbing, and root planing) and/or prosthodontic therapy (fixed or removable dentures based on Eichner classification). For those needing both, periodontal treatment preceded prosthodontic therapy. The CG received lifestyle guidance only, with dental treatment delayed by 3 months for ethical reasons.

**Figure 1:**
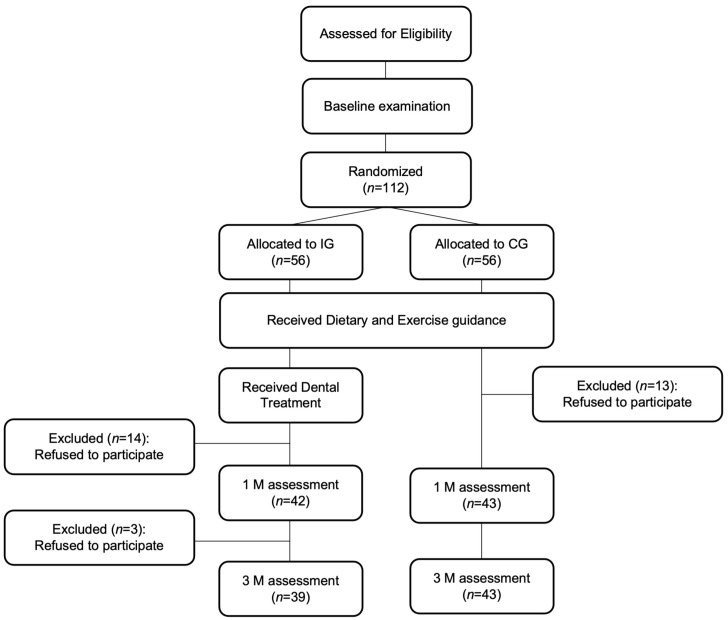
Flowchart of participants throughout the study. Abbreviations: 1 M, 1 month after the study period; 3 M, 3 months after the study period; CG, control group; IG, intervention group.

The sample size calculation was based on the expected difference in waist circumference reported by Doke et al. (2021) [23]. To detect a difference of 3.0 cm with a standard deviation of 5.0 cm, a significance level of 0.05, and a power of 0.80, 40 participants per group were needed. Considering the retention rate of 80%, a target of 50 participants per group was initially estimated. While a priori power calculation was not performed specifically for the OHRQoL outcomes (GOHAI and OHIP-14), the final analyzed sample of 82 participants was used for post hoc power considerations. Of 112 initially enrolled MetS patients, 82 completed the study (IG: 39, CG: 43) after 30 dropped out (Figure 1) [23].

### 2.2. Measurements

The GOHAI and OHIP-14 were selected to provide a multi-dimensional assessment. While GOHAI can be more sensitive to functional limitations and pain in older or medically compromised populations, OHIP-14 can be superior for capturing psychological and social impacts [30]. Using both can allow for a comprehensive understanding of how dental intervention impacts MetS patients’ daily lives.

OHRQoL was assessed at baseline (BL), 1 M, and 3 M using Japanese versions of the GOHAI and OHIP-14. Scores were calculated additively. To ensure data integrity, investigators verified questionnaire completeness immediately upon submission. Both instruments utilized mixed positive and negative items to minimize response bias. (Table 1).

GOHAI is a 12-item survey designed to examine OHRQoL in older individuals. It assessed oral health-related functional, psychological, pain, and discomfort factors [31]. The response options ranged from 1 to 5, where higher scores indicated higher OHRQoL.

The OHIP questionnaire originally included 49 items [32], which assessed dysfunction, pain, and impairment associated with oral conditions [33]. OHIP-14, a shorter variant, was later developed with comparable reliability and validity [34]. The response options ranged from 0 to 4, where higher scores indicated lower OHRQoL.

### 2.3. Statistical Methods

Baseline between-group differences were assessed using Student’s *t*-test. The primary outcome of the study was the waist circumference, while OHRQoL was analyzed as a secondary outcome. Between-group differences for each outcome were initially analyzed using one-way analysis of covariance (ANCOVA). To evaluate the trajectory of OHRQoL scores across the three time points (baseline, 1 month, and 3 months) within each group, repeated-measures analysis of variance (ANOVA) was employed. To control for type I error inflation due to multiple comparisons, post hoc pairwise tests were performed using the Bonferroni correction. The effect sizes for both ANOVA and ANCOVA models were reported using partial eta squared ($\eta_{p}^{2}$). Subgroup analyses were performed using ANCOVA for patients with missing teeth and those with periodontitis alone to examine potential variations based on dental status.

To determine the independent effect of the intervention on OHRQoL while accounting for potential confounders, a multivariable ANCOVA was conducted for the 3-month outcomes. In this model, the 3-month OHRQoL scores were the dependent variables with the study group as the fixed factor, adjusted for baseline OHRQoL scores, age, sex, and baseline waist circumference.

Additionally, Pearson correlation coefficients (r) were calculated to examine the relationship between physical health markers (weight and waist circumference) and OHRQoL scores (GOHAI and OHIP-14), analyzing both absolute values and longitudinal changes (Δ).

All statistical analyses were performed using SPSS Version 25 (IBM Corp., Armonk, NY, USA). The threshold for statistical significance was set at *p* < 0.05.

## 3. Results

### 3.1. Baseline Characteristics

There were no significant differences between the intervention group (IG) and the control group (CG) across any subcategories of the GOHAI or OHIP-14 at baseline. As shown in Table 2, the baseline total GOHAI score was 43.6 ± 15.9 for the IG and 47.5 ± 10.9 for the CG (*p* = 0.196), while the baseline total OHIP-14 score was 10.1 ± 12.6 for the IG and 11.0 ± 11.2 for the CG (*p* = 0.735).

### 3.2. Between-Group Comparisons (Table 3 and Table 4)

Adjusted analysis (ANCOVA) showed that the dental intervention significantly improved key OHRQoL domains compared to the control group.

For GOHAI, while total scores showed a positive trend (*p* < 0.10), the psychosocial domain was significantly superior in the intervention group at both 1 month (Adjusted *p* = 0.008) and 3 months (Adjusted *p* = 0.010) with a moderate effect size ($\eta_{p}^{2}$ ≈ 0.10).

The OHIP-14 results showed broader significance. The intervention group achieved significantly lower (better) total scores at 1 month (Adjusted *p* = 0.036) and 3 months (Adjusted *p* = 0.020). This improvement was driven by high-impact gains in psychological disorders, handicap, and social disability (all *p* ≤ 0.003). Notably, social disability at 3 months demonstrated a large effect size ($\eta_{p}^{2}$ = 0.148, *p* < 0.001), reflecting a substantial clinical benefit in social interaction. No significant differences were found in functional limitations or physical pain domains.

**Table 3:** Comparisons in OHRQoL from OHIP and GOHAI-14 questionnaires between the control (*n* = 43) and intervention (*n* = 39) groups before and after treatment.

		Baseline		1 M			3 M		
		Mean		Mean		*p*-Value *	Mean		*p*-Value *
		Control	Intervention	Control	Intervention		Control	Intervention	
GOHAI	Total score	47.5 (10.9)	43.6 (15.9)	50.1 (10.4)	46.4 (18.2)	0.098	50.2 (10.4)	46.0 (18.0)	0.080
	Functional aspects Q1–5	20.2 (4.7)	18.6 (7.0)	21.2 (4.5)	19.3 (7.7)	0.650	20.9 (4.7)	18.9 (7.6)	0.500
	Psychosocial aspects Q6, 7, 9–11	19.3 (5.3)	17.7 (6.8)	20.5 (5.0)	19.3 (7.8)	0.012 *	21.0 (4.7)	19.3 (7.6)	0.005 *
	Pain & discomfort Q8, 12	8.0 (1.9)	7.3 (2.8)	8.4 (1.9)	7.8 (3.0)	0.369	8.2 (1.9)	7.8 (3.2)	0.359
OHIP-14	Total score	11.0 (11.2)	10.1 (12.6)	8.1 (8.1)	6.1 (11.5)	0.018 *	7.6 (8.8)	6.0 (11.8)	0.010 *
	Functional limitations Q1, 2	1.3 (1.7)	0.9 (1.8)	1.0 (1.4)	0.7 (1.6)	0.325	1.0 (1.5)	0.9 (1.8)	0.262
	Pain Q3, 4	2.6 (2.1)	2.3 (2.3)	2.1 (1.9)	1.4 (2.1)	0.968	1.9 (1.9)	1.3 (2.0)	0.572
	Psychological discomfort Q5, 6	2.4 (2.3)	2.2 (2.4)	1.8 (1.9)	1.2 (1.9)	0.229	1.6 (1.9)	1.1 (1.9)	0.406
	Physical disability Q7, 8	1.4 (1.7)	1.6 (2.4)	1.1 (1.3)	0.9 (2.0)	0.230	1.1 (1.4)	0.8 (2.0)	0.074
	Psychological disorders Q9, 10	1.5 (1.8)	1.3 (2.0)	1.0 (1.3)	0.9 (1.9)	0.002 *	1.1 (1.4)	0.9 (2.1)	0.001 *
	Social disability Q11, 12	0.8 (1.6)	0.6 (1.4)	0.4 (0.9)	0.4 (1.3)	0.024 *	0.4 (0.9)	0.3 (1.3)	0.000 *
	Handicap Q13, 14	0.9 (1.7)	1.1 (1.9)	0.5 (0.9)	0.5 (1.7)	0.001 *	0.6 (1.0)	0.6 (1.8)	0.000 *

Data are presented as mean (standard deviation) (for all such values). Statistical analysis using analysis of covariance (ANCOVA) *; statistically significant *p*-values for between-group differences determined by ANCOVA with baseline values as a covariate. Abbreviations: 1 M, 1 month post-treatment; 3 M, 3 months post-treatment; GOHAI, Geriatric Oral Health Assessment Index; OHIP, Oral Health Impact Profile; OHRQoL, oral health-related quality of life.

**Table 4:** Between-group comparisons of OHRQoL at 1 month and 3 months (adjusted analysis).

		Time	Control	Intervention	95% CI	Adjusted *p*-Value	Effect Size ($\eta_{p}^{2}$)
GOHAI	Psychosocial aspects	1 M	20.5 (5.0)	19.3 (7.8)	[−3.18, −0.62]	0.008 *	0.102
		3 M	21.0 (4.7)	19.3 (7.6)	[−3.12, −0.58]	0.010 *	0.095
OHIP-14	Total score	1 M	8.1 (8.1)	6.1 (11.5)	[−2.95, −0.42]	0.036 *	0.071
		3 M	7.6 (8.8)	6.0 (11.8)	[−2.81, −0.39]	0.020 *	0.082
	Psychological disorders	1 M	1.0 (1.3)	0.9 (1.9)	[−0.31, −0.04]	0.003 *	0.110
		3 M	1.1 (1.4)	0.9 (2.1)	[−0.35, −0.05]	0.002 *	0.125
	Social disability	1 M	0.4 (0.9)	0.4 (1.3)	[−0.15, −0.01]	0.002 *	0.090
		3 M	0.4 (0.9)	0.3 (1.3)	[−0.18, −0.02]	<0.001 *	0.148

Values are mean ± SD; *p*-values are derived from ANCOVA with baseline values as a covariate and adjusted for multiple comparisons using the Bonferroni correction. 95% CI = confidence interval for the mean difference. $\eta_{p}^{2}$ (partial eta squared) represents the effect size: 0.01 (small), 0.06 (medium), and 0.14 (large). Significant values (*p* < 0.05) are indicated in asterisk.

### 3.3. Within-Group Changes (Table 5)

Within-group analysis revealed significant improvements in both groups, though the intervention group (IG) showed more extensive gains across the sub-domains.

**Table 5:** Comparisons of changes in OHRQoL from OHIP and GOHAI-14 questionnaires within the control (*n* = 43) and intervention (*n* = 39) groups before and after treatment.

	Metric	Baseline	1 Month	3 Month	*p*-Value	$\eta_{p}^{2}$	Pairwise
Intervention Group	GOHAI Total	43.6 ± 15.9	46.4 ± 18.2	46.0 ± 18.0	0.094	0.06	N.S.
	GOHAI Functional	18.6 ± 7.0	19.3 ± 7.7	18.9 ± 7.6	0.578	0.014	N.S.
	GOHAI Psychosocial	17.7 ± 6.8	19.3 ± 7.7	19.3 ± 7.6	0.008	0.119	BL < 3 M (*p* = 0.047)
	GOHAI Pain/Discomfort	7.3 ± 2.8	7.8 ± 3.0	7.8 ± 3.2	0.158	0.047	N.S.
	OHIP-14 Total	10.1 ± 12.6	6.1 ± 11.5	6.0 ± 11.8	<0.001	0.185	BL > 1 M (*p* = 0.008), BL > 3 M (*p* = 0.006)
	OHIP Functional Limit	0.9 ± 1.8	0.7 ± 1.6	0.9 ± 1.8	0.54	0.016	N.S.
	OHIP Physical Pain	2.3 ± 2.3	1.4 ± 2.0	1.3 ± 2.0	<0.001	0.195	BL > 1 M (*p* = 0.005), BL > 3 M (*p* = 0.004)
	OHIP Psych Discomfort	2.2 ± 2.3	1.2 ± 1.9	1.1 ± 1.9	<0.001	0.24	BL > 1 M (*p* = 0.001), BL > 3 M (*p* = 0.001)
	OHIP Physical Disability	1.6 ± 2.4	0.9 ± 2.0	0.8 ± 2.0	0.004	0.138	BL > 3 M (*p* = 0.014)
	OHIP Psych Disability	1.3 ± 2.0	0.9 ± 1.9	0.9 ± 2.1	0.044	0.079	N.S.
	OHIP Social Disability	0.6 ± 1.4	0.4 ± 1.3	0.3 ± 1.3	0.115	0.055	N.S.
	OHIP Handicap	1.1 ± 1.9	0.5 ± 1.7	0.6 ± 1.8	0.005	0.131	BL > 1 M (*p* = 0.026)
Control Group	GOHAI Total	47.5 ± 10.9	50.1 ± 10.4	50.2 ± 10.4	0.021	0.088	N.S.
	GOHAI Functional	20.2 ± 4.7	21.2 ± 4.6	20.9 ± 4.7	0.093	0.055	N.S.
	GOHAI Psychosocial	19.3 ± 5.3	20.5 ± 5.0	21.0 ± 4.7	0.012	0.1	BL < 3 M (*p* = 0.034)
	GOHAI Pain/Discomfort	8.0 ± 1.9	8.4 ± 1.9	8.2 ± 1.9	0.137	0.046	N.S.
	OHIP-14 Total	11.0 ± 11.2	8.1 ± 8.1	7.6 ± 8.8	0.022	0.087	N.S.
	OHIP Functional Limit	1.3 ± 1.7	1.0 ± 1.4	1.0 ± 1.5	0.266	0.031	N.S.
	OHIP Physical Pain	2.6 ± 2.1	2.1 ± 1.9	1.9 ± 1.9	0.017	0.092	N.S.
	OHIP Psych Discomfort	2.4 ± 2.3	1.8 ± 1.9	1.6 ± 1.9	0.005	0.117	BL > 3 M (*p* = 0.020)
	OHIP Physical Disability	1.4 ± 1.7	1.1 ± 1.3	1.1 ± 1.4	0.187	0.039	N.S.
	OHIP Psych Disability	1.5 ± 1.8	1.0 ± 1.3	1.1 ± 1.4	0.079	0.059	N.S.
	OHIP Social Disability	0.8 ± 1.6	0.4 ± 0.9	0.4 ± 0.9	0.114	0.05	N.S.
	OHIP Handicap	1.0 ± 1.7	0.5 ± 0.9	0.6 ± 1.0	0.122	0.049	N.S.

Statistical significance for within-group changes over time (Baseline, 1 Month, and 3 Month) was determined using repeated-measures ANOVA. All pairwise comparisons were adjusted for multiple testing using the Bonferroni correction. Abbreviations: GOHAI, Geriatric Oral Health Assessment Index; OHIP, Oral Health Impact Profile; OHRQoL, oral health-related quality of life; $\eta_{p}^{2}$, partial eta squared; N.S., not significant.

In the IG, the OHIP-14 total score improved significantly from baseline to 3 months (10.1 ± 12.6 to 6.0 ± 11.8; *p* < 0.001, $\eta_{p}^{2}$ = 0.185), with significant reductions appearing as early as 1 month (*p* = 0.008). Significant improvements were also noted in OHIP-14 sub-domains including physical pain, psychological discomfort, physical disability, and handicap (all *p* ≤ 0.005). Improvement in the GOHAI total score did not reach significance (*p* = 0.094); however, the psychosocial domain showed a statistically significant increase (*p* = 0.008). In the control group (CG), significant improvements were observed in the GOHAI total score (*p* = 0.021) and the GOHAI psychosocial domain (*p* = 0.012). For OHIP-14, although the total score and physical pain improved over time (*p* < 0.05), pairwise comparisons failed to reach significance after Bonferroni adjustment. The only OHIP-14 sub-domain with a significant pairwise improvement in the CG was psychological discomfort at 3 months (*p* = 0.020).

### 3.4. Subgroup Analysis

#### 3.4.1. Participants with Missing Teeth (Table 6)

For the missing teeth subgroup, the adjusted between-group analysis (Table 6) revealed significant OHRQoL improvements in the intervention group (IG) compared to the control group (CG). The IG demonstrated a substantial reduction in the GOHAI total scores at both 1 month (adjusted MD = −14.80, adjusted *p* = 0.006) and 3 months (adjusted MD = −15.80, adjusted *p* = 0.006) with large effect sizes ($\eta_{p}^{2}$ = 0.292). Within the GOHAI domains, the pain and discomfort as well as psychosocial aspects remained significant after Bonferroni correction (*p* < 0.05).

Similarly, the OHIP-14 total score showed a significant therapeutic advantage for the IG (adjusted *p* = 0.008). While all sub-domains favored the intervention, the most pronounced improvements were observed in psychological disorders, handicap, and social disability, the latter of which displayed the largest effect size in this subgroup at 3 months ($\eta_{p}^{2}$ = 0.447, adjusted *p* < 0.001). Conversely, differences in functional limitations and physical pain were non-significant after adjustment, despite moderate effect sizes.

**Table 6:** Comparisons in OHRQoL from OHIP and GOHAI-14 questionnaires within the control (*n* = 16) and intervention (*n* = 13) groups before and after treatment of those with missing teeth.

		Time	Mean Diff	95% CI	Adjusted *p*-Value	$\eta_{p}^{2}$
GOHAI	Total score	1 M	−14.8	[−27.70, −1.90]	0.006 *	0.292
		3 M	−15.8	[−28.44, −3.16]	0.006 *	0.292
	Functional aspects Q1–5	1 M	−6.6	[−12.26, −0.94]	0.168	0.108
		3 M	−7.6	[−13.06, −2.14]	0.206	0.096
	Psychosocial aspects Q6, 7, 9–11	1 M	−5.6	[−11.43, 0.23]	0.020 *	0.229
		3 M	−5.5	[−11.27, 0.27]	0.004 *	0.312
	Pain & discomfort Q8, 12	1 M	−2.6	[−4.77, −0.43]	0.046 *	0.183
		3 M	−2.7	[−4.98, −0.42]	0.044 *	0.186
OHIP-14	Total score	1 M	−3.6	[−13.89, 6.69]	0.008 *	0.277
		3 M	−3.1	[−13.67, 7.47]	0.008 *	0.277
	Functional limitations Q1, 2	1 M	−0.4	[−1.89, 1.09]	0.134	0.120
		3 M	−0.7	[−2.20, 0.80]	0.110	0.132
	Pain Q3, 4	1 M	−0.8	[−2.56, 0.96]	0.638	0.037
		3 M	−0.6	[−2.41, 1.21]	0.460	0.054
	Psychological discomfort Q5, 6	1 M	−1.1	[−2.86, 0.66]	0.180	0.105
		3 M	−0.9	[−2.60, 0.80]	0.240	0.089
	Physical disability Q7, 8	1 M	−0.1	[−1.89, 1.69]	0.106	0.134
		3 M	0.0	[−1.78, 1.78]	0.076	0.154
	Psychological disorders Q9, 10	1 M	−0.4	[−2.07, 1.27]	0.002 *	0.346
		3 M	−0.6	[−2.31, 1.11]	0.002 *	0.346
	Social disability Q11, 12	1 M	−0.7	[−1.77, 0.37]	0.004 *	0.312
		3 M	−0.4	[−1.58, 0.78]	<0.001 *	0.447
	Handicap Q13, 14	1 M	−0.2	[−1.59, 1.19]	0.002 *	0.346
		3 M	0.0	[−1.46, 1.46]	0.002 *	0.346

Values are mean different. *p*-values are derived from ANCOVA with baseline values as a covariate and adjusted for multiple comparisons using the Bonferroni correction. 95% CI = confidence interval for the mean difference. $\eta_{p}^{2}$ (partial eta squared) represents the effect size: 0.01 (small), 0.06 (medium), and 0.14 (large). Significant values (*p* < 0.05) are indicated in asterisk.

#### 3.4.2. Participants with Periodontitis Only (Table 7)

Adjusted between-group analysis for the periodontal subgroup revealed no significant differences in total OHRQoL scores at either follow-up (Table 7). At 3 months, the GOHAI (MD = 1.60, adjusted *p* = 0.892) and OHIP-14 (MD = −0.30, adjusted *p* = 0.694) total scores remained comparable between the intervention group (IG) and control group (CG).

While most domains showed negligible changes ($\eta_{p}^{2}$: 0.001–0.028), the IG demonstrated significantly better raw scores in the OHIP-14 sub-domains of psychological disorders (*p* = 0.034) and handicap (*p* = 0.043); however, after Bonferroni correction, these differences—along with a marginal trend in social disability (adjusted *p* = 0.092)—did not reach the threshold for statistical significance. Overall, the OHRQoL impact in this subgroup was markedly less pronounced than in the missing teeth subgroup.

**Table 7:** Comparisons in OHRQoL from OHIP and GOHAI-14 questionnaires within the control (*n* = 27) and intervention (*n* = 26) groups before and after treatment of those with periodontitis.

		Time	Mean Diff	95% CI	Adjusted *p*-Value	$\eta_{p}^{2}$
GOHAI	Total score	1 M	−0.4	[−3.88, 3.08]	1.000	0.009
		3 M	1.6	[−1.53, 4.73]	0.892	0.012
	Functional aspects Q1–5	1 M	0.2	[−1.48, 1.88]	0.936	0.011
		3 M	0.3	[−1.25, 1.85]	0.568	0.023
	Psychosocial aspects Q6, 7, 9–11	1 M	−0.4	[−1.76, 0.96]	0.716	0.017
		3 M	1.4	[−0.02, 2.82]	0.474	0.028
	Pain & discomfort Q8, 12	1 M	−0.2	[−0.94, 0.54]	1.000	0.000
		3 M	−0.2	[−0.92, 0.52]	1.000	0.001
OHIP-14	Total score	1 M	−0.6	[−4.80, 3.60]	0.928	0.011
		3 M	−0.3	[−4.14, 3.54]	0.694	0.018
	Functional limitations Q1, 2	1 M	0.1	[−0.54, 0.74]	1.000	0.000
		3 M	0.2	[−0.55, 0.95]	0.760	0.015
	Pain Q3, 4	1 M	−0.1	[−0.99, 0.79]	0.930	0.011
		3 M	0.1	[−0.77, 0.97]	1.000	0.002
	Psychological discomfort Q5, 6	1 M	0.0	[−0.83, 0.83]	1.000	0.001
		3 M	0.0	[−0.78, 0.78]	1.000	0.002
	Physical disability Q7, 8	1 M	0.1	[−0.84, 1.04]	1.000	0.007
		3 M	0.0	[−0.77, 0.77]	1.000	0.002
	Psychological disorders Q9, 10	1 M	−0.1	[−0.90, 0.70]	0.570	0.023
		3 M	−0.2	[−0.86, 0.46]	0.340	0.037
	Social disability Q11, 12	1 M	0.2	[−0.28, 0.68]	1.000	0.008
		3 M	0.2	[−0.20, 0.60]	0.092	0.077
	Handicap Q13, 14	1 M	0.1	[−0.47, 0.67]	1.000	0.004
		3 M	0.2	[−0.32, 0.72]	0.232	0.049

Values are mean different. *p*-values are derived from ANCOVA with baseline values as a covariate and adjusted for multiple comparisons using the Bonferroni correction. 95% CI = confidence interval for the mean difference. $\eta_{p}^{2}$ (partial eta squared) represents the effect size: 0.01 (small), 0.06 (medium), and 0.14 (large).

### 3.5. Multivariable Analysis of OHRQoL Outcomes

To evaluate the independent factors influencing OHRQoL at the 3-month follow-up, a multivariable ANCOVA was performed, adjusting for demographics (age and sex) and baseline metabolic status (waist circumference) (Table 8).

For the GOHAI model, the baseline GOHAI score was the strongest independent predictor of the 3-month outcome (B = 0.46, *p* < 0.001). Age was also found to be a significant covariate (B = −0.17, *p* = 0.018), indicating that older participants tended to report lower OHRQoL scores at follow-up when other factors were kept constant. Similarly, in the OHIP-14 model, baseline scores (B = 0.38, *p* < 0.001) and age (B = 0.18, *p* = 0.031) were significantly associated with the 3-month results. In both models, the study group assignment did not show a significant independent effect (*p* > 0.05) once these baseline and demographic factors were accounted for. Furthermore, while baseline waist circumference was included as a representative metabolic syndrome component, it was not a significant independent predictor of the final OHRQoL scores in this adjusted model (*p* > 0.05).

### 3.6. Correlation Between Metabolic Improvement and OHRQoL

**Figure 2:**
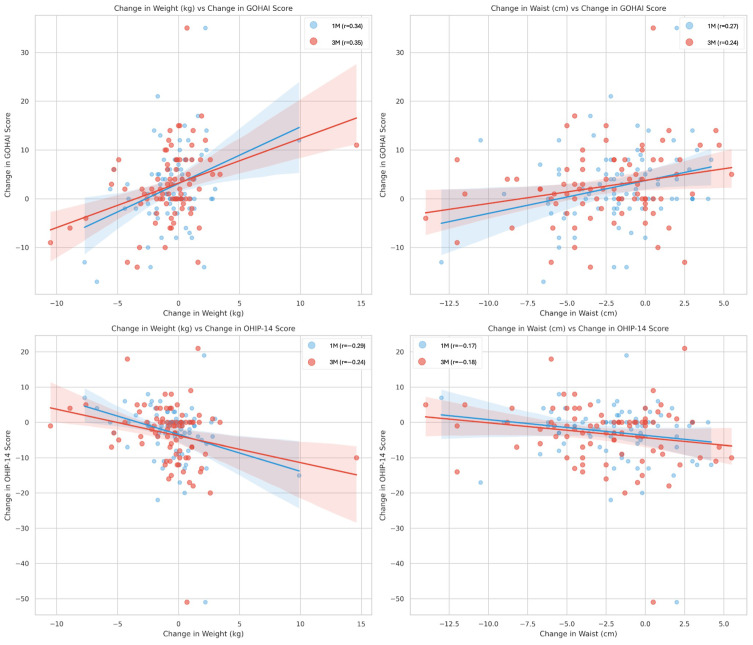
Correlation between longitudinal changes (Δ) in metabolic markers and OHRQoL scores. Solid lines represent the linear regression trends for 1 month (blue) and 3 months (red), with shaded areas indicating the corresponding 95% confidence intervals.

Pearson correlation analyses were performed to examine the relationship between changes (Δ) in physical health markers and OHRQoL scores at both 1 and 3 months (Figure 2).

At the 3-month follow-up, significant positive correlations were observed between the improvement in GOHAI scores and the reductions in body weight (r = 0.355, *p* = 0.001) and waist circumference (r = 0.238, *p* = 0.031). These findings indicate that significant reductions in body weight and waist circumference were associated with higher gains in oral health-related quality of life.

Conversely, OHIP-14 scores showed a significant negative correlation with weight reduction after 3 months (r = −0.244, *p* = 0.027), reflecting that a decrease in body weight was associated with a decrease in oral health impact (representing an improvement in quality of life). Although the correlation between Δwaist circumference and ΔOHIP-14 followed a similar negative trend, it did not reach statistical significance at the 3-month mark (r = −0.176, *p* = 0.114).

## 4. Discussion

This was the first study to compare two major questionnaires on OHRQoL in relation to objectively measured long-term oral function in MetS patients in Japan. This study aimed to evaluate the OHRQoL of such patients using GOHAI and OHIP-14 in terms of oral health. The null hypothesis was rejected correctly based on oral interventions that improved OHRQoL in these patients.

Significant between-group differences in Table 3 and Table 4 highlighted a shift in the intervention group’s psychosocial profile. The large effect size in social disability ($\eta_{p}^{2}$ = 0.148) at 3 months suggested dental intervention facilitated social reintegration by restoring aesthetic and functional confidence. The reductions in the psychological distress and handicap domains indicated that resolving oral disease mitigated the emotional burdens of metabolic syndrome (MetS). While functional domains remained comparable between the groups, the intervention group’s superiority in social and psychological metrics underscored dental care as a non-elective component of holistic MetS management. These findings aligned with Polish [35] and Japanese [36] studies reporting similar psychosocial impacts, despite the variations in functional reporting elsewhere [33].

Although both groups showed minor GOHAI improvements, the intervention group reached statistical significance in psychosocial aspects, reflecting a substantial relief from oral health burdens. Similarly, the OHIP-14 results favored the intervention group in psychological and social categories, suggesting that dental treatment enhanced well-being by mitigating the social impacts of disease [37]. Inconsistencies between the GOHAI and OHIP-14 results reflected the distinct perspectives of each tool, as their use was validated by high EMPRO performance scores (71 and 68, respectively) for older populations [30]. However, a limitation of these self-reported measures is their susceptibility to “response shift,” where patients’ internal standards for quality of life change following treatment, potentially masking the full extent of clinical improvement [30,33]. While the GOHAI effectively identified pain-related dysfunction [38]. The OHIP-14 captured psychological and social disabilities more acutely [33], aligning with Locker’s model where social impacts occurred less frequently than functional issues [33,39].

Within-group analysis (Table 5) demonstrated that dental intervention directly alleviated functional and psychological burdens in MetS patients. The intervention group (IG) showed highly significant improvements in OHIP-14 total scores (*p* < 0.001), specifically through reduced physical pain and psychological discomfort. These results corroborated research linking dietary adherence to improved psychosocial outcomes [40]. Since MetS management required high fiber and lean protein intake, the significant reduction in the physical pain (*p* < 0.001) and handicap (*p* = 0.005) domains suggested that treatment successfully removed physical barriers to healthy eating. Enhanced masticatory function increased patient self-efficacy, as reflected in improved GOHAI psychosocial (*p* = 0.008) and OHIP-14 psychological disability scores. In contrast, the control group (CG) showed no significant changes in physical handicap or disability domains. This disparity suggested that, without active dental restoration, a “nutritional barrier” remained, limiting the psychosocial benefits of lifestyle modifications. Consequently, our findings indicated a synergistic relationship where dental rehabilitation acted as a critical enabler for the dietary changes necessary to manage MetS, ultimately enhancing holistic quality of life.

The relationship between oral health and systemic well-being is complex, as metrics do not always correlate uniformly with OHRQoL [41]. The results from the missing teeth subgroup (Table 6) have reinforced that while lifestyle guidance can improve subjective health, professional dental care is essential to resolve functional and social handicaps associated with tooth loss [39]. Our findings aligned with evidence that prosthetic status and masticatory function significantly impacted the physical index of health-related QoL [42,43,44]. Specifically, reducing pain and discomfort addressed a critical barrier to systemic health management; as seen in the literature, denture wearers can often avoid essential food groups due to difficulty chewing [45]. By resolving these limitations, the intervention likely facilitated better adherence to MetS dietary modifications, positioning dental treatment as a prerequisite for successful metabolic therapy.

Conversely, the periodontal subgroup (Table 7) showed no significant between-group differences in total OHRQoL, reflecting the “silent” nature of periodontal disease. While oral hygiene instructions can improve both periodontal and metabolic markers [22], the CG’s unexpectedly better scores in the psychological domains at 3 months may stem from temporary post-treatment sensitivity in the IG. Despite modest changes, our results broadly aligned with studies linking periodontal care to improved OHRQoL [12,13,14], likely by reducing the systemic inflammation that can exacerbate MetS [17]. Thus, integrating periodontal care with lifestyle guidance remains vital for optimizing long-term outcomes.

Subgroup analysis also revealed a divergence between instruments, as the intervention significantly improved OHIP-14 scores, while the CG maintained higher GOHAI scores in the missing teeth group. This inconsistency likely stems from the GOHAI’s higher sensitivity to functional pain versus the OHIP-14’s focus on broader psychosocial impacts [33]. However, the small sample size of these subgroups (*n* = 13–16) limits statistical power; consequently, these findings should be interpreted as exploratory.

Multivariable ANCOVA (Table 8) revealed that while the study group was not an independent predictor, baseline OHRQoL and age emerged as the most significant factors influencing the final scores. The significance of age as a covariate suggested that age-related dental changes, such as cumulative periodontal damage, may modulate the degree of improvement achievable through lifestyle interventions alone [46]. Notably, the lack of an independent effect for baseline waist circumference—contrasted with the significant correlation between change (Δ) in waist circumference and OHRQoL—highlighted a key clinical distinction: OHRQoL outcomes were dictated more by the success of the weight-loss process than by initial metabolic severity.

Consequently, a central finding of this study was the robust link between metabolic improvement and OHRQoL enhancement. Correlation analysis (Figure 2) showed that greater reductions in body weight and waist circumference were significantly associated with improved OHRQoL, particularly with GOHAI scores. This relationship strengthened between the 1-month and 3-month marks, suggesting that sustained weight management was a primary driver of subjective oral health improvements. As abdominal obesity has been a hallmark of metabolic syndrome [47], these results have implied that metabolic interventions can extend beyond systemic markers to positively influence a patient’s perceived functional and psychosocial well-being [48].

This study has several limitations. The 3-month follow-up may not fully capture the long-term sustainability of OHRQoL improvements, particularly for periodontal therapy. Additionally, the sample size was powered for waist circumference rather than OHRQoL; therefore, while a post hoc power analysis (d = 0.6, power ≈ 0.78) can suggest the study was sufficiently powered for primary outcomes, the small subgroup sizes (*n* = 13–16) could limit the ability to detect significant differences in all sub-domains. Consequently, the subgroup results should be interpreted as exploratory. The absence of a sensitivity analysis to evaluate the consistency of results across different metabolic phenotypes or the influence of outliers further limits our understanding of how MetS severity variations can affect OHRQoL. Future studies with larger cohorts should utilize multivariable models to further isolate the effects of dental intervention and integrate broader evaluations—such as physical activity, stress, and sleep quality—with dental therapy assessments to better understand the holistic impact on MetS patients.

In conclusion, integrated dental and lifestyle interventions significantly improved OHRQoL in MetS patients, primarily by enhancing psychosocial well-being and social reintegration. While age and baseline OHRQoL were significant independent predictors of the 3-month outcomes, the magnitude of improvement was directly driven by metabolic success. Specifically, significant correlations between reductions in body weight and waist circumference and improved GOHAI and OHIP-14 scores indicated that OHRQoL was functionally linked to systemic weight management. These findings suggested that addressing the “nutritional barrier” through dental rehabilitation, while targeting measurable anthropometric goals, is essential for the holistic management of patients with metabolic syndrome.

## Figures and Tables

**Table 1:** Categories and subcategories of GOHAI and OHIP-14.

GOHAI	OHIP-14
Functional aspects	Functional limitations
Limits kinds or amounts of food	Trouble pronouncing words
Had trouble biting/chewing food	Worsened sense of taste
Uncomfortable to swallow	Pain
Prevented from speaking	Painful aching in mouth
Discomfort when eating	Uncomfortable eating foods
Psychological aspects	Psychological discomfort
Limits contact with others	Felt self-conscious
Unhappy with appearance	Felt tense
Worried or concerned	Physical disability
Nervous or self-conscious	Diet has been unsatisfactory
Uncomfortable eating in front of people	Had to interrupt meals
Pain & discomfort	Psychological disorders
Use medication to relieve pain	Difficult to relax
Teeth and gums sensitive to hot/cold	Felt embarrassed
	Social disability
	Felt irritable with others
	Totally unable to function
	Handicap
	Felt life is less satisfying
	Difficulty doing usual jobs

Abbreviations: GOHAI, Geriatric Oral Health Assessment Index; OHIP, Oral Health Impact Profile.

**Table 2:** Baseline characteristics.

		Group		Total	*p*-Value
		Control (*n* = 43)	Intervention (*n* = 39)		
GOHAI	Total score	47.5 (10.9)	43.6 (15.9)	45.6 (13.6)	0.196
	Functional aspects Q1–5	20.2 (4.7)	18.6 (7.0)	19.4 (5.9)	0.210
	Psychosocial aspects Q6, 7, 9–11	19.3 (5.3)	17.7 (6.8)	18.5 (6.1)	0.247
	Pain & discomfort Q8, 12	8.0 (1.9)	7.3 (2.8)	7.7 (2.4)	0.191
OHIP-14	Total score	11.0 (11.2)	10.1 (12.6)	10.6 (11.9)	0.735
	Functional limitations Q1, 2	1.3 (1.7)	0.9 (1.8)	1.1 (1.7)	0.427
	Pain Q3, 4	2.6 (2.1)	2.3 (2.3)	2.5 (2.2)	0.540
	Psychological discomfort Q5, 6	2.4 (2.3)	2.2 (2.4)	2.3 (2.3)	0.644
	Physical disability Q7, 8	1.4 (1.7)	1.6 (2.4)	1.5 (2.1)	0.666
	Psychological disorders Q9, 10	1.5 (1.8)	1.3 (2.0)	1.4 (1.9)	0.635
	Social disability Q11, 12	0.8 (1.6)	0.6 (1.4)	0.7 (1.5)	0.544
	Handicap Q13, 14	0.9 (1.7)	1.1 (1.9)	1.0 (1.8)	0.704

Data are presented as mean (standard deviation) (for all such values). Statistical analysis using Student’s *t*-test.

**Table 8:** Multivariable ANCOVA for OHRQoL Scores at 3 months.

Dependent Variable	Independent Variable	B	SE	t-Value	*p*-Value
**GOHAI** (3 months)	(Constant)	29.7	11.91	2.49	0.015
	Group (Intervention vs. Control)	−0.32	1.37	−0.23	0.819
	Baseline GOHAI Score	0.46	0.08	5.55	<−0.001 *
	Age	−0.17	0.07	−2.42	0.018 *
	Sex (Male vs. Female)	−2.41	1.46	−1.65	−0.102
	Baseline Waist Circumference (cm)	0.13	0.09	1.45	0.152
**OHIP**-14 (3 months)	(Constant)	−3.78	11.48	−0.33	0.743
	Group (Intervention vs. Control)	0.01	1.58	0	0.997
	Baseline OHIP-14 Score	0.38	0.08	4.62	<0.001 *
	Age	0.18	0.08	2.2	0.031 *
	Sex (Male vs. Female)	2.46	1.72	1.43	0.156
	Baseline Waist Circumference (cm)	−0.06	0.1	−0.62	0.54

B, unstandardized coefficient; SE, standard error; *t*, *t*-statistic. * Statistically significant at *p* < 0.05. Waist circumference was selected as the primary covariate representing metabolic status to avoid multicollinearity with body weight, as it was the primary inclusion criterion for the study.

## Data Availability

The original contributions presented in the study are included in the article; further inquiries can be directed to the corresponding author.

## Abbreviations

The following abbreviations are used in this manuscript:

1 M	1 Month Post-Intervention
3 M	3 Months Post-Intervention
ANCOVA	One-Way Analysis of Covariance
ANOVA	Analysis of Variance
BL	Baseline
CG	Control Group
HRQoL	Health-Related Quality of Life
IG	Intervention Group
MetS	Metabolic Syndrome
NCEP-ATP III	Adult Treatment Panel of the National Cholesterol Education Program
OHIP-14	Oral Health Impact Profile-14
OHRQoL	Oral Health-Related Quality of Life
QoL	Quality of Life
RCT	Randomized Controlled Trial
TFAS	Total Fitness Analysis System
TMDU	Tokyo Medical and Dental University
EMPRO	Evaluating Measures of Patient-Reported Outcomes
